# The Effect of Multi-Strategy Nutrition Education Programs on Hedonic Hunger and Nutrition Status in Adolescents

**DOI:** 10.3390/children11101188

**Published:** 2024-09-28

**Authors:** Osman Bozkurt, Hilal Yildiran

**Affiliations:** 1 Department of Nutrition and Dietetics, Faculty of Health Sciences, Erzurum Technical University, Erzurum 25050, Turkey; 2 Department of Nutrition and Dietetics, Faculty of Health Sciences, Gazi University, Emek Bişkek Cad. 6, Sokak, Ankara 06490, Turkey; hilalciftci@gazi.edu.tr

**Keywords:** multi-strategy nutrition education programs, adolescent, children’s power of food scale 9, hedonic hunger, nutritional status

## Abstract

*Objective:* Increasing the awareness of adolescents about hedonic hunger, understanding the difference between homeostatic hunger and hedonic hunger, and adolescents learning to control themselves to stop excessive food consumption are extremely important for a healthy adulthood. The study aimed to evaluate the effects of the multi-strategy nutrition education programs (MSNEP) on hedonic hunger, food addiction, nutrition literacy, and nutritional status in adolescents. *Methods:* This study was planned using a pre-test and post-test design. The MSNEP was conducted with 132 adolescents (11–15 years; 69 boys, 63 girls) for 4 weeks (45 min–1 h/session). Data were obtained using questionnaires with face-to-face interviews at pre-education (baseline) and post-education (week 4 and week 8). The survey form included sociodemographic information, nine item short version of Children’s Power of Food Scale (C-PFS-9), the Yale Food Addiction Scale for Children 2.0 (YFAS-C 2.0), the Adolescent Nutrition Literacy Scale (ANLS), anthropometric measurements, and 24-hour dietary recall. *Results:* A decrease in C-PFS-9 total scores was found compared to the baseline (*p* < 0.001). While the YFAS-C 2.0 score decreased in boys compared to the baseline (*p* < 0.05), no significant difference was found in girls (*p* > 0.05). A difference was found in the ANLS scores for girls (*p* = 0.01), but no difference was found in the scores for boys during the study (*p* > 0.05). At week 4, the consumption of dairy products, legumes, vegetables and fruits, bread and grains, nuts, and hard-shelled seeds increased compared to the baseline (*p* < 0.05). Also, daily protein and fiber intake increased (*p* < 0.05). Accordingly, a higher YFAS-C 2.0 score predicted greater hedonic hunger. A lower ANLS score was a predictor for higher food taste and food available scores. *Conclusions:* In conclusion, the MSNEP was found to have positive effects on hedonic hunger, food addiction, nutritional literacy, and healthy eating behaviors. The study revealed differences between girls and boys. In order to maintain healthy body weights in adolescents, it is recommended that the MSNEP be provided in schools.

## 1. Introduction

The physical hunger of cells in the body is defined as homeostatic hunger [1]. Hedonic hunger is one of the types of hunger that affects the brain centers associated with pleasure and reward [2]. Unlike homeostatic hunger, hedonic hunger creates a solid drive to consume palatable foods [3]. The smell, flavor, and texture directly affect food intake. These factors may affect appetite control, leading to food intake for pleasure, even when not physically hungry [4]. Neural systems play an essential role in regulating hedonically induced food intake. Palatable foods have been observed to elicit a more significant response in the neural region of the brain [5].

Although hormonal, genetic, and environmental factors (socioeconomic development, urbanization, decreased physical activity, the easy accessibility of palatable foods with high energy density, etc.) play a role in the development of obesity, the main factor in the formation of a predisposition to obesity is excessive food consumption. Even if there is no energy deficit, the frequent consumption of palatable foods with high energy density causes the food to be seen as a reward [6,7]. The ease of access to energy-dense and palatable foods, or even the thought of the food being present, activates dopaminergic reward pathways in the brain [8,9]. Palatable foods have been shown to trigger dopaminergic circuits and instinctive eating in rodents [10]. It is stated that the reward center activity in the brains of individuals with a high desire to eat is higher. Therefore, it is suggested that any functional disorder in this part of the brain increases the obesity risk [11]. This explains the lower levels of striatal dopamine D2 receptors in obese individuals compared to non-obese individuals [12]. Furthermore, sensory cues that occur before eating palatable foods suggest that the food is seen as a reward [13,14]. Furthermore, the hedonic urge to eat triggers an intense craving for fatty and high-energy foods. The continued high consumption of such foods activates the addiction factor. Food addiction, which is characterized by both emotional and physiological effects, can lead to uncontrollable eating behavior [13]. In a study of university students, it was found that a relationship between food addiction and hedonic hunger stems from the need to consume highly palatable foods [15].

In adolescents, conscious control is less strong than in adults, and they live in an obesogenic environment, which increases unhealthy food preferences and food consumption for pleasure [16]. Understanding the processes that influence food choices during this period is essential for physical and mental health in adulthood. High consumption of unhealthy foods and beverages in adolescents is associated with increased dietary energy intake, increased body weight in adulthood, and a greater risk of chronic disease [16]. The determination of hedonic hunger markers in adolescence is thought to provide a better understanding of the eating disorders and body weight gain that may occur in this period [17]. Also, studies investigating the effects of nutritional literacy on food choices in adolescents are increasing [15,18]. Nutritional literacy is the degree to which individuals read and understand the nutrition information necessary to acquire nutrition skills and make appropriate nutrition decisions. In order to reduce the increasing prevalence of nutrition-related health problems, it is of great importance to increase the level of knowledge of individuals and society about nutrition and to develop healthy eating skills and behaviors [15].

There is a limited number of studies on adolescents and hedonic hunger conducted in Turkey [19,20]. Increasing the awareness of adolescents about hedonic hunger, understanding the difference between homeostatic hunger and hedonic hunger, and learning to control themselves to stop excessive food consumption is extremely important for a healthy adulthood. Therefore, it is important to develop and implement nutrition education programs for adolescents to gain healthy eating habits and to create mindful eating. The most effective factors that ensure success in nutrition education intervention studies for adolescents are reported to be behavior change strategies, education and training strategies, adequate duration and intensity, the participation of peers and parents, self-evaluation, and the provision of feedback [21,22]. A systematic review evaluated the effects of multi-strategy nutrition education programs (programs that include training on healthy nutrition, healthy food and beverage choices, healthy food preparation techniques, and physical activity, with the use of various educational techniques) on adolescent health and nutrition [22]. Although the contents, training techniques, and durations of nutrition education in these programs varied, four studies reported significant changes in the anthropometric measurements of adolescents and nine studies reported significant changes in dietary intake in a systemic review [22]. The study aimed to evaluate the effects of a multi-strategy nutrition education programs (MSNEP) on hedonic hunger, food addiction, nutrition literacy, and nutritional status in adolescents.

## 2. Methods

### 2.1. Study Design and Participants

This study was planned using a pre-test and post-test design. It was carried out in schools, with adolescents aged 11–15. Participants were selected by simple random sampling from randomly selected schools in Erzurum (one of the metropolitan cities of Turkey). The sample size was 36, with the G Power analysis (G*Power 3.0.10) showing a 0.25 effect size, 90% power probability, and 5% type 1 error level. Adolescents with no chronic and psychological issues, who did not need any special diet, and who agreed to join the study were included in the research. The study started with 168 adolescents but was completed with 132 adolescents.

The multi-strategy nutrition education programs (MSNEP) was developed by the researchers. In this program, adolescents were given training at school for 45 min to 1 h per week for 4 weeks; also, home activity assignments were given. To observe the effectiveness of the 4-week training results, measurements were retaken at the end of the 8th week. Three evaluations were made: pre-test (baseline) and post-test (week 4 and week 8). However, anthropometric measurements were taken at the baseline and in the 8th week.

Ethical permission was obtained from the Erzurum Technical University Ethics Committee (Meeting Number: 5; Decision Number: 1; 15 February 2021) in addition to the Erzurum Provincial Directorate of National Education (16 March 2021). The study was carried out following the principles outlined in the Helsinki Declaration. Written informed consent was obtained from the parents and the verbal consent of the children was also obtained.

### 2.2. The Multi-Strategy Nutrition Education Programs (MSNEP)

In this study, by examining the educational content, strategies, and materials used in the studies in the literature, a multi-strategy nutrition education programs (MSNEP) was prepared, with the aim of increasing the level of healthy nutrition knowledge and literacy, developing healthy eating behaviors, increasing mindful eating, increasing physical activity levels, and reducing hedonic hunger and food addiction in adolescents. Increasing the awareness and discrimination of hunger and satiety cues enhances the body’s natural ability to self-regulate food consumption without reducing the enjoyment of food. Each session lasts between 45 min and 1 h. The training was conducted at the school once a week in the activity hall; 20–30 min of the lessons were conducted as interactive lectures and 20–30 min as in-class activities. In order to reinforce the information learned in the course, homework assignments were given. The detailed content of the MSNEP is presented in detail in Table 1.

### 2.3. Measures

Data were obtained using the survey and face-to-face interviews. The survey form included sociodemographic information, dietary habits, nine item short version of Children’s Power of Food Scale (C-PFS-9), the Yale Food Addiction Scale for Children 2.0 (YFAS-C 2.0), the Adolescent Nutrition Literacy Scale (ANLS), anthropometric measurements, and 24-hour dietary recall.

#### 2.3.1. The Children’s Power of Food Scale

The Children’s Power of Food Scale (C-PFS) was developed to evaluate hedonic hunger, which refers to individuals’ cognitive and emotional responses toward food and eating when there is no physiological requirement for dietary energy [23]. The 15-item C-PFS developed by Lowe et al. (2006) was adapted into a nine-item short version of C-PFS-9 by Stone et al. (2020) [23,24]. The Turkish reliability and validity study of C-PFS-9 was conducted by Bozkurt and Yildiran (2024) [25]. The C-PFS-9 consists of three subscales: food available (FA, i.e., food readily available in the environment but not physically present), food present (FP, i.e., food that is physically present in the environment but not tasted), and food tasted (FT, i.e., food that has been tasted but not consumed). The Cronbach’s α coefficient of the scale, consisting of the FA, FP, and FT sub-scales, was found to be 0.90, 0.88, and 0.86, respectively. The scale was prepared according to the five-point Likert system, with the responses scored as follows: “1 = Do not agree at all; 2 = Agree a little; 3 = Agree somewhat; 4 = Agree; 5 = Strongly agree”. The scores of the items in each sub-scale were added and the arithmetic mean was calculated (divided by the number of items). Higher scores indicated greater hedonic hunger [23].

#### 2.3.2. Yale Food Addiction Scale for Children 2.0 (YFAS-C 2.0)

The Yale Food Addiction Scale (YFAS) was developed by Gearhardt et al. (2009) [26]. It was adapted for use with children as YFAS-C 2.0 by Schiestl et al. (2018) [27]. The Turkish validity and reliability study of YFAS-C 2.0 was conducted by Yılmaz in 2018 [28]. It evaluated the state of eating in the form of cravings during the last 12 months in children and, thus, food addiction. In this scale, foods with addictive potential are divided into four groups. There are 16 items prepared according to the DSM-5 diagnostic criteria that question children’s thoughts about these foods and their experiences in the last year. While the Cronbach’s α coefficient of the original scale developed by Gearhardt et al. was 0.90, it was found to be 0.901 in the Turkish version [26,28]. The scale is prepared according to the five-point Likert system and the responses scored as follows: “0 = Never; 1 = Rarely; 2 = Sometimes; 3 = Very often; 4 = Always”. This scale consists of a single factor and, as the scale score increases, the level of food addiction increases [28].

#### 2.3.3. Adolescents’ Nutrition Literacy Scale

The Adolescents’ Nutrition Literacy Scale (ANLS) was developed by Bari [29], and the Turkish validity and reliability of the scale were analyzed by Türkmen et al. in 2017 [30]. The scale consists of 22 items and 3 sub-scales. Seven items constitute the sub-scales of Functional Nutrition Literacy, six items constitute the sub-scales of Interactive Nutrition Literacy, and nine items constitute the sub-scales of Critical Nutrition Literacy. In the Turkish version of the scale, the Cronbach’s α coefficient of the sub-scales was found to be 0.66, 0.71, and 0.84, respectively, and the total Cronbach’s α coefficient was found to be 0.80. [30]. The scale prepared according to the five-point Likert system scores the responses as follows: “1 = I do not agree at all; 2 = I do not agree; 3 = I am undecided; 4 = I agree; 5 = I completely agree”. The minimum score on the scale is 22 points and the maximum score is 110 points, and, as the total score increases, nutritional literacy also increases [30].

#### 2.3.4. Dietary Intake

One-day food consumption records (24-hour dietary recall) were taken from the participants at pre-test (baseline) and post-test (week 4 and week 8). The “Meal and Food Photo Catalog” was used to determine the sizes and amounts of the food and beverages consumed [31]. The contents of the meals consumed by participants were calculated using a “Standard Recipes” book [32]. The dietary energy and nutrients in the meals were analyzed using the “Nutrition Information Systems Package Program” (BeBiS, Ebispro for Windows, Germany; Turkish Version/BeBiS 9.0) [33]. Individual daily intakes of nutrients were compared with the dietary reference intakes (DRIs) as categorized by age and sex. Food groups (dairy products, meat, chicken, fish, eggs, legumes, nuts and hard-shelled seeds, bread and grains, sugar and sugary foods, vegetables and fruits) were evaluated.

#### 2.3.5. Anthropometric Measurements

Measurements regarding the children’s height, weight, and waist circumference were conducted by the researcher following the techniques described by Lohman et al. (1988) [34]. The anthropometric measurements of the children were taken at the baseline and at the eighth week. The World Health Organization (WHO) (2007) growth standards and the WHO AnthroPlus software (version 1.0.4, February 2011) program were applied to evaluate the weight, height, body mass index (BMI), and Z-scores. The measurements were categorized according to the Z-score junctions [35].

#### 2.3.6. Data Analysis

The data were analyzed using SPSS 23.0. Since the quantitative data did not meet the assumption of normal distribution, nonparametric hypothesis tests were used. For continuous quantitative variables, the mean, standard deviation (SD), the median and interquartile range (IQR) were obtained, while for categorical variables, interpretations were obtained by giving frequencies and percentages. The Mann–Whitney or Kruskal–Wallis tests were applied to find value differences. The Friedman test was performed for the analysis of repeated measures. To identify correlations between parameters, Spearman’s correlation analysis was used, A multiple linear regression model was used to identify independent predictors of hedonic hunger. The results were evaluated at the 95% confidence interval, at a *p* < 0.05 significance level.

## 3. Results

A total of 132 adolescents (69 males and 63 females) participated in the study, with a median (IQR) age of 14.0 (1.0) years (range 11–15 years). The mothers of 43.9% of the participants were primary school graduates, 26.9% were university graduates, and 21.3% were high school graduates. When the educational status of the fathers was analyzed, it was seen that 40.9% were high school graduates and 25.0% were university graduates. While the mothers of 86.4% of the children were not working, the fathers of 84.8% were working (Table 2).

The median and IQR values of C-PFS-9, YFAS-C 2.0, and ANLS total scores and subscales during the study period according to gender are given in Table 3. The total C-PFS-9 score of the adolescents before the training was determined as 2.7 (1.3). At the same time, the total C-PFS-9 score of the girls was 2.7 (1.1), which was 2.4 (1.1) higher than the score of the boys (*p* = 0.019). A decrease in the week 4 and week 8 C-PFS-9 total scores was found in both boys and girls compared to the baseline (*p* < 0.001). Only the difference between FT scores according to gender was significant (*p* < 0.001). It was found that the FA and FP scores decreased in boys compared to the baseline (*p* < 0.05), and there was no significant difference in FT scores (*p* > 0.05). In girls, FP and FT scores were found to have decreased compared to the baseline (*p* < 0.05) and there was no significant difference in FA scores (*p* > 0.05).

While the baseline score of adolescents for YFAS-C 2.0 was 15.5 (16.0), a decrease was found in week 4 (12.8 (18.0)) and week 8 (13.4 (23.0)) (*p* < 0.05). While the YFAS-C 2.0 score decreased in boys compared to the baseline (*p* < 0.05), no significant difference was found for girls (*p* > 0.05). While the ANLS score was 66.0 (12.8) at baseline, there was a significant increase in the score (67.0 (12.0)) at week 4 (*p* = 0.01). Although a similar difference was found in the ANLS scores of girls (*p* = 0.01), no difference was found in the scores for boys during the study (*p* > 0.05) (Table 3).

**Table 3:** The values of the C-PFS-9, YFAS-C 2.0, and ANLS total scores and sub-scales during the study period, grouped according to gender.

Scales and Their Sub-Scales	Boys (n = 69)	Girls (n = 63)	Total (n = 132)	*p* β
	Median (IQR)	Median (IQR)	Median (IQR)	
C-PFS-9 Total Score				
Baseline	2.4 (1.1) a	2.7 (1.1) a	2.7 (1.3) a	**0.019**
Week 4	2.0 (1.5) b	2.1 (1.6) b	2.0 (1.5) b	0.383
Week 8	2.2 (1.6) b	2.4 (1.9) b	2.3 (1.8) b	0.074
*p* *	**<0.001**	**<0.001**	**<0.001**	
Food Available				
Baseline	2.2 (1.5) a	2.0 (1.7)	2.0 (1.3) a	0.248
Week 4	1.9 (1.5) b	2.0 (1.3)	2.0 (1.2) b	0.539
Week 8	2.0 (2.0) a,b	2.0 (2.0)	2.0 (2.0) a,b	0.196
*p* *	**0.035**	0.368	**0.022**	
Food Present				
Baseline	2.7 (1.2) a	3.0 (1.7) a	3.0 (1.0) a	0.267
Week 4	2.3 (1.8) b	2.3 (1.3) b	2.3 (1.7) b	0.252
Week 8	2.0 (1.8) b	2.7 (1.3) b	2.3 (1.6) b	0.141
*p* *	**0.002**	**0.025**	**0.001**	
Food Tasted				
Baseline	2.3 (1.7)	3.3 (1.7) a	2.7 (1.7)	**<0.001**
Week 4	2.0 (1.3)	2.7 (2.0) b	2.0 (1.7)	**0.004**
Week 8	2.0 (1.7)	2.7 (2.3) b	2.3 (1.7)	**0.023**
*p* *	0.096	**<0.001**	**<0.001**	
YFAS-C 2.0 Total Score				
Baseline	14.7 (14.5) a	16.3 (18.0)	15.5 (16.0) a	0.182
Week 4	11.7 (16.0) b	13.9 (18.0)	12.8 (18.0) b	0.418
Week 8	12.3 (23.5) b	14.6 (20.0)	13.4 (23.0) b	0.462
*p* *	**0.013**	0.792	**0.034**	
ANLS Total Score				
Baseline	66.0 (12.0)	66.0 (16.0) a	66.0 (12.8) a	0.690
Week 4	66.0 (9.0)	70.0 (14.0) b	67.0 (12.0) b	**0.022**
Week 8	66.0 (9.5)	69.0 (14.0) a,b	67.0 (12.8) a,b	**0.024**
*p* *	0.183	**0.001**	**0.001**	

β Mann–Whitney U test; * Friedman test. The same letters in columns indicate no statistical difference. IQR: Interquartile range. C-PFS-9: Children’s Power of Food scale—9 items. YFAS-C 2.0: Yale Food Addiction Scale for Children 2.0. ANLS: Adolescents’ Nutrition Literacy Scale. Bold values indicate that *p* < 0.05.

At the baseline, the median BMI value for boys was 21.0 (4.8) kg/m^2^ and for girls was 19.3 (4.0) kg/m^2^. While 51.5% of the participants (boys 42.0%; girls 61.9%) were in the normal classification according to body mass index-for-age z-score (BAZ), 36.4% (boys 43.5%; girls 28.6%) were in the overweight/obese classification. In boys, at week 8, compared to baseline, the body weight, BMI, and BAZ score decreased (*p* < 0.05), while height and height-for-age z-score (HAZ) increased (*p* > 0.05). In girls, the height and HAZ scores increased (*p* < 0.05) (Table 4).

The daily intake of food groups of adolescents during the study period is given in Table 5. After sessions (week 4), the consumption of dairy products, legumes, vegetables and fruits, bread and grains, nuts, and hard-shelled seeds increased compared to the baseline (*p* < 0.05). At the baseline, the consumption of meat, chicken, and fish, when grouped according to gender, was lower in girls (*p* < 0.05). After the session (week 4 and week 8), the consumption of nuts and hard-shelled seeds, vegetables, and fruits was found to be lower in boys than in girls (*p* < 0.05). There was no difference in the intake of eggs, legumes, dairy products, bread and grains, sugar, and sugary foods between genders (*p* > 0.05).

The daily energy and macronutrient intake levels of adolescents and their DRI-meeting percentages throughout the study are presented in Table 6. The median and IQR values of total dietary daily energy intake at the baseline were 1843.6 (560.7) kcal, 1911.1 (354.8) kcal at week 4, and 2013.6 (524.1) kcal at week 8 (*p* < 0.05). Daily protein intake increased in week 4 (69.4 (21.8) g) and week 8 (71.8 (25.4) g) compared to the baseline (60.6 (26.9) g) (*p* < 0.05). Dietary fiber was higher in week 4 (20.9 (11.9) g) and week 8 (24.3 (22.8) g) than at the baseline (15.4 (8.3) g) (*p* < 0.05). After the sessions (week 4 and week 8), fiber intake was found to be higher in girls (23.7 (14.7) g) than in boys (19.2 (10.8) g), and this difference was found to be significant (*p* < 0.05).

The C-PFS-9 total score showed a significant positive correlation with the YFAS-C 2.0 score (baseline rs = 0.620; *p* < 0.001; week 4 rs = 0.524; *p* < 0.001; week 8 rs = 0.575; *p* < 0.001). Also, positive relationships were found between the C-PFS-9 subscales and the YFAS-C 2.0 score (*p* < 0.05). There was no significant association between ANLS and C-PFS-9 (*p* > 0.05). However, statistically significant negative correlations were found between the ANLS and the subscales of the C-PFS-9.

Table 7 indicates the predictors of hedonic hunger at the baseline (all models are adjusted for sex, age, dietary energy intake, and BMI z-score). Accordingly, a higher YFAS-C 2.0 score (β = 0.032, *p* < 0.001) predicted higher hedonic hunger. A lower ANLS score was a predictor of higher FT and FA scores.

## 4. Discussion

Problematic eating habits among children/adolescents may have adverse emotional and physical results, emphasizing the importance of promoting beneficial interventions. In this study, MSNEP was found to reduce hedonic hunger scores, reduce food addiction scores (only boys), and increase ANLS scores (only girls) in adolescents. In boys, at week 8, the BAZ score decreased and the HAZ score increased, while in girls, an increase in the HAZ score was observed. After sessions (week 4), the consumption of dairy products, legumes, vegetables and fruits, bread and grains, nuts, and hard-shelled seeds increased compared to the baseline. Also, daily protein and fiber intake increased. Accordingly, a higher YFAS-C 2.0 score predicted higher hedonic hunger, while a lower ANLS score was a predictor of higher FT and FA scores.

### 4.1. Evaluation of the Effects of MSNEP on Hedonic Hunger and the Factors Related to Hedonic Hunger

The most effective factors that ensure success in nutrition education intervention studies for adolescents are reported to be behavior change strategies, education strategies, sufficient duration, the participation of peers and parents, self-evaluation, and the provision of feedback [21,22]. A systematic review evaluated the effects of multi-strategy nutrition education programs (programs that include education on healthy nutrition, healthy food and beverage choices, healthy food preparation techniques, physical activity, and various educational techniques) on adolescent health and nutrition. Although the content, training techniques, and duration of nutrition education in these programs vary, four studies reported significant changes in the anthropometric measurements of adolescents, and nine studies reported significant changes in dietary intake. It is stated that, especially, the inclusion of school personnel, teachers, and families in these education programs, as well as the provision of an environment that encourages healthy nutrition in schools, can have significant effects on adolescent nutrition [22]. The literature shows that success in nutrition education interventions is achieved with behavior change-based approaches rather than a knowledge-based approach [22]. In our study, it is thought that the decrease in hedonic hunger scores was achieved due to the provision of sessions emphasizing healthy food choices, increasing eating awareness, the participation of peers and parents, self-evaluation, and feedback.

Gender is a factor that affects the concentrations of estradiol, which affects the hypothalamus region that regulates appetite and eating behavior. A higher estradiol concentration in women indicates that they may have less hunger and more satiety [36]. However, some studies have found that women have higher levels of hedonic hunger and the desire to reward themselves with food [37,38,39]. A study of Iranian adults found that hedonic hunger was higher in women [37]. Similarly, Japanese women have been found to have higher hedonic hunger scores than men [40]. Conversely, the appearance of foods, as well as their taste, affects adolescents’ food choices [41]. The FT subscale score of C-PFS-9 and total C-PFS-9 score were found to be higher in the girls who participated, compared to the boys. Similarly, in a study conducted on adolescents, FT scores were found to be higher in girls [20]. These results support the idea that gender and food addiction may create a greater hedonic effect. To the best of our knowledge, no study in the literature evaluates the relationship between nutrition literacy and hedonic hunger in adolescents. The high level of nutritional literacy in individuals suggests that they will be able to make more conscious food choices in the future.

### 4.2. Evaluation of the Effects of MSNEP on Food Addiction

Adolescents were found to view sugary foods as a reward to a greater extent than adults. This shows that adolescents may be more prone to food addiction [42]. Mindful eating interventions to promote healthy eating behaviors in adolescents are generally studies aimed at achieving bodyweight loss with diet and increasing eating awareness [43,44,45]. Such interventions may also include nutrition education by a dietitian that includes healthy eating behaviors [45]. Within the scope of this study, in order to gain healthy eating behaviors, mindfulness education (the environmental factors affecting food intake, emotional factors, awareness of hunger, and satiety signals), as well as education on healthy nutrition, were provided. In this study, the decrease in YFAS-C 2.0 scores in girls after the sessions was not statistically significant, while the decrease in boys was significant. In a study conducted in adults (n = 923), the food addiction score (YFAS) was found to be higher in women than in men [46]. In a study evaluating food addiction in adolescents, it was found that girls had higher YFAS scores than boys [47]. In another study conducted on adolescents (11–18 years), the YFAS scores of girls were found to be higher than boys but this was not statistically significant [48]. In this study, the girls’ YFAS scores were found to be higher than the boys’, but it was not statistically significant. At the same time, the relationship between high YFAS scores and female gender and high hedonic hunger scores was determined in this study. There is a need for further research into the finding that YFAS-C 2.0 scores did not decrease in girls after the sessions.

### 4.3. Evaluation of the Effects of MSNEP on Nutrition Literacy

Nutrition literacy is the ability of individuals to read and understand nutrition information. A study evaluating the effects of nutrition education on the level of nutrition knowledge in adolescents found that the education program increased the level of nutrition knowledge in adolescents. In addition, inadequate nutrition knowledge was associated with unhealthy eating habits [49]. In a study conducted in adolescents, the ANLS score was significantly higher in girls than in boys [50]. In a similar study, the ANLS score was found to be higher in adolescent girls compared to boys [51]. This study found a significant difference between baseline and week 4 ANLS scores in girls. However, this difference was not significant at week 8. There was no difference in the boys’ scores. This result suggests that the nutrition literacy information provided within the scope of the training may have been received with more interest by female adolescents.

### 4.4. Evaluation of the Effects of MSNEP on Nutritional Status

The World Health Organization defines adolescence as a period of rapid growth and development and social and psychological changes between the ages of 10 and 19 [52]. Physical change in adolescents is observed mainly in terms of height. The fastest age at which height growth is observed in girls is 12 years (9 cm/year; 0.75 cm/month), and the fastest age at which height growth is observed in boys is 14 years (10.3 cm/year; 0.86 cm/month) [53]. A study evaluated the effectiveness of a sports nutrition intervention designed to improve body composition in 25 high-school male athletes over 12 weeks. They suggested that targeted nutritional interventions positively impact adolescent athletes’ body composition [54].

In a nutrition education program conducted by Dewar et al. in 2013 with adolescents to prevent obesity, training was given on healthy eating, healthy eating behavior, and healthy food preparation techniques. Clinically significant improvements were found in adolescent body fat ratios 24 months after the intervention study [55]. In our study, while the BAZ score decreased in boys, the HAZ score increased, and the HAZ score also increased in girls. Growth is rapid in this age group, and an increase in height was observed in adolescents, as expected, during the study period. Studies with a more extended intervention period and those including only obese adolescents would be of interest.

Within the scope of the MSNEP, the effects of hedonic hunger and food addiction on nutritional status were explained to adolescents; training was given on healthy nutrition, and practical applications were made. Food groups were explained in the sessions, and their effects on health were mentioned. Hunger and satiety signals were emphasized, and it was explained that hedonic hunger can cause unhealthy food preferences and excess energy intake. The healthy food plate was explained, and practical applications were demonstrated on how the participants could create a healthy plate. After the training, the consumption of milk and products, legumes, vegetables and fruits, bread and cereals, oil seeds, and nuts increased compared to the baseline. Daily protein intake and fiber intake also increased after the sessions.

A study showed a significant increase in vegetable and fruit consumption in the adolescents’ fourth month after the internet-based nutrition education program [56]. In a nutritional intervention study conducted on adolescents in Canada, an increase in milk, vegetable, and fruit intake was observed. However, it was found that favorable improvements in food selection disappeared following the 10th week after the intervention [57]. In a study on Indonesian female adolescents, a multi-strategy nutrition intervention included healthy eating/food choices and behavior change education [58]. After the intervention, the consumption of vegetables and fruits increased, and the consumption of sugary drinks decreased [58]. While the positive effects of MSNEP on the vegetable and fruit consumption of adolescents were found, its effects on sugary food consumption were limited in terms of evaluation. Daily consumption of sugary foods was found to be low in our study group, so it could not be evaluated.

Although there were significant improvements in scale scores and dietary intake after the training (week 4), some values returned to baseline values in week 8. Considering this situation, extending the duration of the training program may result in more permanent behavior changes.

The study had some limitations. First, the MSNEP period could have been more extended. A more long-term program may contribute to some lasting eating behavior changes in children. Due to the restrictions during the COVID-19 pandemic period, the assessment of physical activity levels was insufficient, and some practical activities could not be implemented sufficiently.

## 5. Conclusions

The MSNEP contributes to decreased hedonic hunger and food addiction and increased nutrition literacy in adolescents. It has been found that this program positively affects the adolescents’ nutritional status. The study revealed differences between girls and boys. Thus, more studies are needed to explain the reasons behind these differences. After the training (week 4), the scale scores and nutritional intake improved significantly. However, some values returned to baseline in week 8. Given this, prolonging the training program may lead to more permanent behavior change. Longitudinal studies with larger samples should be conducted to analyze the impact of this program on body weight in overweight or obese children. In order to maintain a healthy body weight in adolescents, it is recommended that MSNEP be provided in schools.

## Figures and Tables

**Table 1:** The content of the multi-strategy nutrition education programs.

Topic	Content	Classroom Activities
Session 1 Health and Nutrition Literacy	Definitions containing fundamental information about health and nutrition were explained. It was emphasized from which professional groups and from which sources reliable and accurate information about health and nutrition can be obtained. In this session, the basic purposes of health and nutrition literacy and why it is important were emphasized. The session specifically outlined what to look for on food and beverage labels. (20–30 min)	A label-reading activity was carried out with adolescents on healthy (for example: dairy products) and unhealthy foods (for example: chips). We encouraged adolescents to develop the habit of reading labels while shopping. An interactive discussion was held in the class regarding important issues to be considered while shopping for food. At the end of this training, the aim was to increase the participants’ health and nutrition literacy levels and to learn how to read food labels. (30 min)
Session 2 Nutrition and Nutrients	Within the scope of this session, fundamental concepts such as food, nutrition types, and adequate and balanced nutrition were included. The primary nutrients were introduced, rich sources of nutrients were identified, and the important functional functions of nutrients were explained. (30 min)	Turkey’s dietary guidelines were introduced. In-class practice was carried out with the adolescents through posters on nutrient-rich food source matching. Practice exercises concerned personal hygiene and food hygiene, and information brochures were distributed. (30 min)
Session 3 Food Groups and Healthy Food Choices	In this session, food groups, to which food groups the foods belong, the recommended daily portion amounts of these food groups according to age and gender, and healthy nutrition were explained, working in line with the recommendations of the Turkey Dietary Guidelines. An example of a healthy food plate was given and how the participants can create a healthy plate was explained. (25 min)	In order to reinforce the information they learned in this session, the students were asked to create a healthy eating plate. Each student was given a healthy eating plate model and was asked to plan their own meals (breakfast/lunch/dinner). (35 min)
Session 4 Mindful Eating and Physical Activity	In this session, the types of hunger were defined, and the concept of hedonic hunger was emphasized. This session was provided to gain an awareness of eating behaviors (environmental factors affecting food intake, awareness of hunger and satiety signals, and emotional factors), especially for ensuring hedonic hunger control. Awareness of the hunger and satiety signals of adolescents was emphasized. It was explained that hedonic hunger can cause unhealthy food preferences and excessive energy intake. In addition, the definition of physical activity, the levels of activities performed during the day, and physical activity recommendations were explained. (30 min)	The scenarios that were created discussed how and what could affect food choices. An activity was conducted on environmental factors that affect portion perception (such as tall thin/short chubby glasses and small/large bowl sizes). In this way, it was shown that environmental factors can cause excessive food consumption. Scenarios were created on how to recognize the hunger and satiety signals given by the body, and an in-class activity was conducted. At the end of the session, brochures were distributed to parents (tips for increasing mindful eating for family members/tips for reducing inactivity in adolescents). (30 min)

**Table 2:** Distribution of children and parents across demographic characteristics (n = 132).

Variables	Number	%
Gender		
Girls	63	47.7
Boys	69	52.3
Child age (years)		
Median (IQR)	14.0 (1.0)	
Mother’s educational status		
Illiterate	3	2.3
Literate	4	3.0
Primary school	58	43.9
Middle School	34	25.8
High school	24	18.2
University	9	6.8
Mother’s working status		
Working	18	13.6
Not working	114	86.4
Father’s educational status		
Illiterate	-	-
Literate	4	3.0
Primary school	23	17.4
Middle School	18	13.6
High school	54	40.9
University	33	25.1
Father’s working status		
Working	112	84.8
Not working	20	15.2

IQR: Interquartile range.

**Table 4:** Anthropometric values of adolescents during the study period, grouped according to gender.

	Boys (n = 69)					Girls (n = 63)				
	Baseline		Week 8		*p* *	Baseline		Week 8		*p* *
	Mean ± SD	Median (IQR)	Mean ± SD	Median (IQR)		Mean ± SD	Median (IQR)	Mean ± SD	Median (IQR)	
Body weight (kg)	54.9 ± 11.43	55.0 (13.9)	54.5 ± 11.0	55.0 (12.8)	**0.046**	49.5 ± 8.30	50.0 (12.0)	49.7 ± 8.28	50.0 (11.0)	0.335
Height (cm)	161.3 ± 7.66	162.0 (10.0)	161.8 ± 7.63	163.0 (11.0)	**<0.001**	157.1 ± 6.73	158.0 (7.5)	157.5 ± 6.82	158.0 (8.0)	**<0.001**
BMI (kg/m^2^)	20.9 ± 3.54	21.0 (4.8)	20.8 ± 3.44	20.8 (4.7)	**<0.001**	20.0 ± 2.91	19.3 (4.0)	19.9 ± 2.85	19.2 (5.0)	0.922
BMI for age z-score	0.7 ± 1.3	0.92 (1.5)	0.6 ± 1.29	0.75 (1.43)	**0.004**	0.2 ± 1.1	0.39 (4.0)	0.2 ± 1.03	0.22 (1.49)	0.913
Height for age z-score	0.4 ± 1.0	0.67 (1.3)	0.5 ± 1.01	0.67 (1.2)	**<0.001**	−0.01 ± 0.97	−0.11 (1.2)	0.1 ± 0.97	−0.05 (1.2)	**<0.001**

* Mann-Whitney U test. BMI: Body mass index. IQR: Interquartile range. Bold values indicate that *p* < 0.05.

**Table 5:** Daily intake of food groups by adolescents during the study period.

Daily Food Group Intakes	Boys (n = 69)		Girls (n = 63)		Total (n = 132)		
	Mean ± SD	Median (IQR)	Mean ± SD	Median (IQR)	Mean ± SD	Median (IQR)	*p* β
Dairy Products (g/day)							
Baseline	124.4 ± 106.1	80.0 (135.0) a	161.4 ± 157.2	130.0 (170.0) a,b	142.9 ± 134.8	100.0 (150.0) a	0.298
Week 4	218.5 ± 180.2	150.0 (240.0) b	216.3 ± 195.9	160.0 (300.0) a	217.4 ± 187.1	160.0 (253.8) b	0.619
Week 8	118.7 ± 96.1	100.0 (120.0) a	145.8 ± 135.9	80.0 (200.0) b	131.60 ± 117.1	100.0 (150.0) a	0.775
*p* *	**0.013**		**0.017**		**<0.001**		
Meat, Chicken, Fish (g/day)							
Baseline	95.9 ± 73.7	100.0 (100.0)	68.5 ± 70.0	40.0 (95.0)	82.4 ± 72.9	62.5 (116.3)	**0.021**
Week 4	105.6 ± 93.2	60.0 (120.0)	85.7 ± 95.7	55.0 (65.0)	96.0 ± 94.5	60.0 (92.5)	0.071
Week 8	119.9 ± 117.6	90.0 (120.0)	65.8 ± 83.7	40.0 (90.0)	94.1 ± 106.0	52.5 (90.3)	**<0.001**
*p* *	0.433			0.114	0.409		
Eggs (g/day)							
Baseline	30.6 ± 39.5	17.0 (50.0)	36.6 ± 43.5	33.0 (50.0)	33.5 ± 41.4	17.0 (50.0)	0.556
Week 4	38.5 ± 48.3	30.0 (50.0)	31.4 ± 31.4	35.0 (50.0)	35.1 ± 41.1	35.0 (50.0)	0.624
Week 8	34.5 ± 46.8	15.0 (50.0)	26.8 ± 36.5	5.0 (20.0)	30.8 ± 42.2	8.0 (50.0)	0.689
*p* *		0.120		0.496	0.151		
Legumes (g/day)							
Baseline	16.2 ± 24.4	0.0 (30.0) a	13.0 ± 21.9	0.0 (20.0) a	14.7 ± 23.2	0.0 (30.0) a	0.581
Week 4	20.4 ± 29.2	0.0 (30.0) a,b	22.2 ± 24.1	15.0 (50.0) a,b	21.3 ± 26.8	6.0 (36,0) a,b	0.449
Week 8	16.8 ± 20.8	4.0 (30.0) b	23.7 ± 27.8	15.0 (48.0) b	20.1 ± 24.6	15.0 (32.0) b	0.159
*p* *	**0.030**			**0.005**	**<0.001**		
Nuts and Hard-shelled Seeds (g/day)							
Baseline	10.8 ± 13.6	3.0 (15.0) a	12.2 ± 15.6	15.0 (18.0) a	11.4 ± 14.5	10.0 (18.0) a	0.532
Week 4	22.5 ± 18.4	23.0 (33.0) b	32.6 ± 22.1	30.0 (30.0) b	27.3 ± 20.82	30.0 (30.0) b	**0.007**
Week 8	16.4 ± 14.8	16.0 (28.0) a,b	24.1 ± 17.7	20.0 (26.0) b	20.1 ± 16.6	16.0 (24.0) c	**0.009**
*p* *	**<0.001**			**<0.001**	**<0.001**		
Bread and Grains (g/day)							
Baseline	267.4 ± 117.1	233.0 (141.0) a	233.7 ± 81.9	223.0 (131.0) a	251.3 ± 102.8	231.5 (125.0) a	0.194
Week 4	254.5 ± 108.4	257.0 (116.0) b	229.6 ± 90.9	225.0 (140.0) b	242.6 ± 100.8	245.5 (125.0) b	0.255
Week 8	325.1 ± 133.6	311.0 (210.5) a	324.5 ± 133.3	315.0 (240.0) a	324.8 ± 132.9	315.0 (225.5) c	0.929
*p* *	**<0.001**			**<0.001**	**<0.001**		
Sugar and Sugary Foods (g/day)							
Baseline	18.5 ± 32.5	1.0 (20.0) a,b	15.4 ± 22.7	0.0 (30.0)	17.0 ± 28.2	0.5 (22.8) a,b	0.797
Week 4	25.9 ± 31.8	10.0 (50.0) a	20.1 ± 27.5	10.0 (45.0)	23.2 ± 29.9	10.0 (45.0) a	0.418
Week 8	15.9 ± 20.6	10.0 (30.0) b	15.9 ± 25.3	0.0 (33.0)	15.9 ± 22.9	0.0 (30.8) b	0.254
*p* *		**0.028**		0.387		**0.034**	
Vegetables and Fruits (g/day)							
Baseline	301.1 ± 405.0	206.0 (263.0) a	316.4 ± 235.4	325.0 (359.0) a	308.4 ± 333.8	260.0 (328.8) a	0.122
Week 4	363.9 ± 310.4	278.0 (300.0) b	437.3 ± 281.3	384.0 (195.0) b	398.9 ± 298.0	339.0 (278.5) b	**0.005**
Week 8	339.0 ± 196.8	336.0 (320.0) b	432.8 ± 236.9	388.0 (326.0) b	383.8 ± 221.1	364.5 (286.3) b	**0.023**
*p* *	**0.002**		**0.001**		**<0.001**		

* Friedman test. β: Mann–Whitney U test. The same letters in columns indicate no statistical difference. IQR: Interquartile range. Bold values indicate that *p* < 0.05.

**Table 6:** Daily energy intake, macronutrient intake, and fiber intake levels of adolescents and DRI-meeting percentages.

Dietary Energy and Macronutrients					DRI %			
Daily Energy and Nutrient Intakes	Boys (n = 69)	Girls (n = 63)	Total (n = 132)		Boys (n = 69)	Girls (n = 63)	Total (n = 132)	
	Median (IQR)	Median (IQR)	Median (IQR)	p^β^	Median (IQR)	Median (IQR)	Median (IQR)	*p* β
Energy (kcal)								
Baseline	1853.1 (594.4) a	1816.1 (498.5) a	1843.6 (560.7) a	0.690	81.3 (26.1)	87.7 (24.1)	82.9 (26.1)	**0.036**
Week 4	1973.5 (404.8) b	1898.0 (315.0) b	1911.1 (354.8) b	0.223	86.6 (17.8)	91.7 (15.2)	89.2 (14.6)	**0.010**
Week 8	2001.7 (479.9) b	2025.01 (541.1) b	2013.6 (524.1) b	0.811	87.8 (21.1)	97.8 (26.1)	92.6 (25.3)	**0.003**
*p* *	**0.026**	**0.001**	**<0.001**					
Protein (g)								
Baseline	64.8 (27.7) a	57.3 (20.4) a	60.6 (26.9) a	0.047	143.2 (63.9)	158.1 (74.2)	147.2 (67.8)	0.807
Week 4	71.0 (25.6) b	67.9 (19.5) b	69.4 (21.8) b	0.109	172.9 (88.6)	171.2 (83.1)	171.8 (84.4)	0.644
Week 8	75.5 (22.7) b	64.1 (24.7) a,b	71.8 (25.4) b	**0.024**	171.8 (85.0)	175.3 (79.7)	172.8 (78.8)	0.829
*p* *	**0.004**	**0.010**	**<0.001**					
Fat (g)								
Baseline	66.7 (38.6) a	76.5 (28.3) a,b	75.9 (36.9) a	0.279				
Week 4	85.6 (31.2) b	81.7 (22.2) a	82.7 (29.9) b	0.917				
Week 8	74.1 (35.9) a	74.5 (32.0) b	74.5 (33.1) a	0.663				
*p* *	**0.007**	**0.002**	**<0.001**					
Carbohydrate (g)								
Baseline	202.6 (106.8) a	213.9 (76.1) a	208.7 (89.2) a	0.690	155.8 (82.2)	164.5 (58.6)	160.5 (68.6)	0.690
Week 4	213.9 (71.9) a	215.4 (64.9) a	214.6 (62.9) a	0.344	164.6 (55.3)	165.6 (49.9)	165.1 (48.4)	0.344
Week 8	247.7 (111.7) b	269.9 (112.1) b	260.3 (108.9) b	0.253	190.5 (85.9)	207.6 (86.2)	200.2 (83.8)	0.253
*p* *	**0.038**	**<0.001**	**<0.001**					
Fiber (g)								
Baseline	15.6 (7.9) a	15.2 (8.7) a	15.4 (8.3) a	0.518	50.4 (25.5)	58.6 (33.4)	54.2 (27.6)	**0.046**
Week 4	19.2 (10.8) a,b	23.7 (14.7) b	20.9 (11.9) b	**0.021**	62.1 (34.7)	91.3 (56.5)	71.6 (44.4)	**<0.001**
Week 8	22.0 (18.3) b	31.2 (25.3) b	24.3 (22.8)	0.072	70.9 (58.9)	120.2 (97.2)	79.9 (82.2)	**<0.001**
*p* *	**0.009**	**<0.001**	**<0.001**					

* Friedman test. The same letters in columns indicate no statistical difference. β: Mann–Whitney U test. Bold values indicate that *p* < 0.05. IQR: Interquartile range.

**Table 7:** Multiple linear regression analysis for hedonic hunger prediction at baseline.

	C-PFS-9 Total Score			Food Tasted Score			Food Available Score			Food Present Score		
	β	*p*	95% CI	β	*p*	95% CI	β	*p*	95% CI	β	*p*	95% CI
YFAS-C 2.0 score	0.032	**<0.001**	0.024, 0.041	0.022	**<0.001**	0.011, 0.033	0.028	**<0.001**	0.016, 0.039	0.031	**<0.001**	0.020, 0.042
ANLS score	−0.004	0.248	−0.008, 0.011	−0.021	**<0.001**	−0.032, 0.030	−0.010	**0.045**	−0.001, 0.019	−0.001	0.765	−0.010, 0.007
	R^2^ = 0.342, *p* < 0.001			R^2^ = 0.285, *p* < 0.001			R^2^ = 0.206, *p* < 0.001			R^2^ = 0.281, *p* < 0.001		

All models were adjusted for sex, dietary energy intake, and BMI z-score. YFAS-C 2.0 score: Yale Food Addiction Scale for Children 2.0; ANLS: Adolescents’ Nutrition Literacy Scale. Bold values indicate that *p* < 0.05.

## Data Availability

The datasets generated and/or analyzed during the current study are not publicly available due to privacy restrictions but are available from the corresponding author upon reasonable request.
